# A multicentric prospective analysis of the management of edentulous jaw fractures in the elderly population: a World Oral Maxillofacial Trauma (WORMAT) project

**DOI:** 10.1007/s10006-026-01575-z

**Published:** 2026-05-26

**Authors:** Fabio Roccia, Michela Omedè, Federica Sobrero, Giulia Cremona, Utku Nezih Yilmaz, Mikail Atabay, Malamatenia Bourazani, Stavros Skiadas, Žiga Kovačič, Nik Krebs, Anamaria Sivrić, Mario Kordić, Gian Battista Bottini, Maximilian Goetzinger, Luis Fernando de Oliveira Gorla, Valfrido Antonio Pereira-Filho, Emil Dediol, Boris Kos, Vitomir S. Konstantinovic, Nikola Miković, Sahand Samieirad, Antonio Mari Roig, Aguilera Padros Ferran, Sean Laverick, Tamay Mehmet, Rodolfo Benech, Maria Taheny DMD, Carlo Strada, Mehul Raiesh Jaisani, Ahmed Gaber Hassanein, Mahmoud Sobhi Allam, Timothy Aladelusi FWACS, Fabio Volpe, Chiara Copelli

**Affiliations:** 1 Division of Maxillofacial Surgery, Città della Salute e della Scienza Hospital, University of Turin, Corso Bramante 88, Turin, 10126 Italy; 2 Department of Oral and Maxillofacial Surgery, Dicle University, Diyarbakir, Turkey; 3 Department of Oral and Maxillofacial Surgery, Hippocratio General Hospital, Athens, Greece; 4 Department of Maxillofacial and Oral Surgery, University Medical Centre, Ljubljana, Slovenia; 5 Clinic for ENT and OMS, University Clinical Hospital, Mostar, Bosnia and Herzegovina; 6 Department of Oral and Maxillofacial Surgery, Center for Reconstructive Surgery, Paracelsus Medical Private University (PMU), Salzburg, Austria; 7 Department of Diagnosis and Surgery, Division of Oral and Maxillofacial Surgery, São Paulo State University, UNESP, Araraquara, São Paulo Brazil; 8 Department of Maxillofacial Surgery, University Hospital Dubrava, Zagreb, Croatia; 9 Clinic of Maxillofacial Surgery, School of Dental Medicine, University of Belgrade, Belgrade, Serbia; 10 Oral and Maxillofacial Diseases Research Center, Mashhad University of Medical Sciences, Mashhad, Iran; 11 Department of Oral and Maxillofacial Surgery, University Hospital of Bellvitge, Barcelona, Spain; 12 Department of Oral and Maxillofacial Surgery, University of Dundee, Dundee, UK; 13 Oral & Maxillofacial Department, St John’s Hospital, Howden Road West, Livingston - Edinburgh, UK; 14 Department Oral and Maxillofacial Surgery, Dharan, Nepal; 15 Maxillofacial Surgery Unit, General Surgery Dpt, Faculty of Medicine, Sohag University, Sohag, Egypt; 16 Department of Oral and Maxillofacial Surgery, College of Medicine, University of Ibadan, Ibadan, Nigeria; 17 Maxillofacial Surgery Unit, Interdisciplinary Department of Medicine, University of Bari, “Aldo Moro”, Bari, Italy

**Keywords:** Prospective, Elderly, Maxillofacial injuries, Edentulous jaw

## Abstract

**Purpose:**

Data on edentulous jaw fractures in elderly patients are limited, particularly for maxillary injuries. This prospective multicentre observational study aimed to evaluate the characteristics, management, and outcomes of edentulous maxillary and mandibular fractures in elderly patients.

**Methods:**

Patients aged ≥ 60 years with edentulous maxillary and/or mandibular fractures admitted to 17 centres participating in the World Oral and Maxillofacial Trauma (WORMAT) project between March 2024 and March 2025 were prospectively enrolled. The following data were collected: age, sex, cause, fracture site and type, grade of bone atrophy, Facial Injury Severity Scale (FISS), associated injuries, surgical approach, type of treatment, use of a bone graft, length of hospitalisation. Clinical outcomes were assessed at 6 weeks and 3 months.

**Results:**

Among 364 elderly patients with maxillofacial trauma, 93 (25.5%) were edentulous and 69 sustained jaw fractures. Falls were the most common mechanism of injury. Maxillary fractures mainly involved the zygomaticomaxillary complex and were frequently managed conservatively, whereas mandibular fractures predominantly affected the body and symphysis/parasymphysis and were treated with open reduction and internal fixation in 89% of cases, usually with rigid fixation. Fracture site displacement was statistically associated with ORIF, both for maxillary (*p* = 0.010) and mandibular fractures (*p* = 0.026). Complications were mainly plate exposure in three patients with mandibular fractures.

**Conclusion:**

Edentulous jaw fractures in the elderly are rare and typically result from low-energy trauma. Displaced fractures, especially of the atrophic mandible, are mainly managed surgically, with a clear tendency to employ rigid fixation.

## Introduction

The progressive aging of the global population, particularly in industrialised countries, driven by declining fertility rates and increased life expectancy, has been accompanied by a proportional rise in traumatic injuries among elderly individuals, including maxillofacial trauma [1–5].

Epidemiological studies on maxillofacial trauma in older adults consistently report a higher prevalence of mid-facial fractures compared to fractures of the lower third of the face. Low-energy mechanisms—most commonly falls—represent the predominant cause of injury [2, 3, 6–8]. With regard to the treatment strategies, several studies have shown that increasing age is associated with a more conservative management approach, with surgical intervention generally reserved for displaced fractures, particularly those of the mandible. This trend has been confirmed by recent population-based analyses, including the study by Kultanen et al. [5].

Despite a growing body of literature on maxillofacial trauma in the elderly, the data specifically addressing the management of edentulous jaw fractures remain limited. Although edentulism does not necessarily imply bone atrophy, progressive alveolar bone resorption—often related to early tooth loss and frequently associated with conditions such as osteoporosis, particularly in elderly women—represents a significant challenge in the treatment of these injuries. In addition, advanced age is commonly associated with multiple comorbidities, which further complicate surgical decision-making and perioperative management for this patient population.

While the surgical management of edentulous mandibular fractures has been described in a limited number of studies—most of which are single-centre, retrospective, or based on small patient cohorts [9–15]—information regarding edentulous maxillary fractures is limited [16, 17].

Therefore, the aim of this prospective multicentre observational study was to analyse the characteristics, treatment modalities, and clinical outcomes of edentulous maxillary and mandibular fractures in elderly patients admitted to 17 centres participating in the World Oral and Maxillofacial Trauma (WORMAT) project [18]. To the best of our knowledge, this study represents the first prospective multicentre investigation specifically focused on edentulous jaw fractures in the elderly population.

## Materials and methods

A total of 17 oral and maxillofacial surgery divisions participating in the WORMAT project, as summarised in Table 1, received an Excel database to collect data consecutively from patients aged ≥ 60 years, who were admitted between 1 March 2024 and 1 March 2025 for maxillofacial fractures. A PDF file with the instructions for its correct compilation was also provided. The participating centres were encouraged to submit preliminary data 3 months after the project’s commencement to allow the research team to verify the accuracy of the database entries [18]. 

**Table 1:** Maxillofacial surgery units participating in the WORMAT project

Country	City	Affiliation
Austria	Salzburg	Dpt. Oral and Maxillofacial Surgery, Paracelsus Medical University
Bosnia and Herzegovina	Mostar	Clinic for ENT and OMS, University Clinical Hospital
Brazil	Araraquara	Dpt. of Diagnosis and Surgery, Division of Oral and Maxillofacial Surgery, São Paulo State University, UNESP
Croatia	Zagreb	Dpt. Maxillofacial Surgery, University Hospital Dubrava
Egypt	Sohag	Maxillofacial Surgery unit, General surgery Dpt, Faculty of Medicine, Sohag University
Greece	Athens	Dpt. Oral and Maxilofacial Surgery, Hippokration General Hospital
Iran	Mashhad	Oral and Maxillofacial Diseases Research Center, Mashhad University of Medical Sciences
Italy	Turin	Division of Maxillofacial Surgery, Città della Salute e della Scienza Hospital
Italy	Bari	Division of Maxillofacial Surgery, Azienda Ospedaliera-Universitaria Consorzio Policlinico di Bari
Nigeria	Ibadan	Dpt. Oral and Maxillofacial Surgery, College of Medicine, University of Ibadan
Nepal	Dharan	Oral and Maxillofacial Unit, B.P. Koirala Institute of Health Sciences
Serbia	Belgrade	Clinic of Maxillofacial Surgery, School of Dental Medicine, University of Belgrade
Slovenia	Ljubljana	Dpt. Maxillofacial and Oral Surgery, University Medical Centre
Spain	Barcelona	Dpt. Oral and Maxillofacial surgery, University hospital of Bellvitge
Turkey	Diyarbakir	Dpt. of Oral and Maxillofacial Surgery, Dicle University
United Kingdom	Livingston-Edinburgh	Oral and Maxilofacial dpt., St John’s Hospital
United Kingdom	Dundee	Dpt. Oral and Maxillofacial Surgery, University of Dundee

The patients for this prospective study were selected based on the following inclusion criteria: a diagnosis of edentulous mandibular and/or maxillary fractures confirmed by a preoperative computer tomography scan, and no history of previous facial trauma. Only patients with complete clinical and radiological records and a minimum follow-up period of 3 months were included.

The following data were collected: age, sex, cause of injury (categorised as road traffic accident [RTA], fall, assault, sport-related injury, work-related injury, or other), fracture site and type (non-displaced, displaced, comminuted), grade of bone atrophy at the fracture site (using the Luhr classification [19] for mandibular fractures and the Cawood & Howell classification [20] for maxillary fractures), Facial Injury Severity Scale (FISS) score [21], associated injuries (classified as orthopaedic, neurological, spinal, ocular, thoracic, or abdominal), surgical approach (for mandibular fractures, divided into intraoral, transbuccal, translesional, or extraoral), type of treatment (observational, closed reduction, open reduction and internal fixation [ORIF]), use of a bone graft, length of hospitalisation, and outcomes (assessed at follow-up visits at 6 weeks and 3 months).

The following outcomes were recorded at follow-up: the need for revision surgery, non-union, soft tissue and bone infections, plate removal, surgical wound infection or dehiscence, hardware loosening or failure, and post-traumatic sensory changes.

Postoperative soft tissue infections were identified by the presence of oedema, induration with erythema, or purulent discharge from the surgical site, with or without hardware exposure. Bone infections were identified based on radiological signs such as osteopenia, bone sequestration, and periosteal reaction.

In patients undergoing ORIF for mandibular fractures, the number and thickness of the plates used were recorded (categorised as ≤ 1.4 mm, 1.5–2 mm, or > 2 mm). The type of osteosynthesis was defined as “non-rigid” when the fracture was fixed with a single plate of thickness ≤ 1.4 mm, and “rigid” when the fracture was fixed with a plate of thickness ≥ 1.5 mm or with at least two plates of any thickness [22–24]. Osteosynthesis was considered mixed when at least one of two or three fractures was treated with rigid osteosynthesis [25].

For zygomaticomaxillary complex (ZMC) fractures, the sites of osteosynthesis were recorded (zygomaticomaxillary buttress, infraorbital rim, frontozygomatic suture, and temporozygomatic suture).

This study was approved by the Institutional Review Board (IRB — protocol 1164/2023) and was conducted in accordance with all the relevant guidelines of the Declaration of Helsinki.

### Statistical Analysis

The association between fracture displacement and the use of ORIF was assessed using Fisher’s exact test, with statistical significance set at *p* < 0.05. The relationship between mandibular atrophy grade and the choice of osteosynthesis plates was evaluated using the chi-square test, with Bonferroni correction applied for multiple comparisons. All analyses were performed using SPSS (version 28.0.1.0).

## Results

During the study period, out of 364 patients aged ≥ 60 years admitted for maxillofacial fractures, 93 (25.5%) patients were edentulous. Among these, 69 patients (74%) had fractures of the maxilla and/or mandible, with 8 patients having fractures involving two sites.

A total of 41 patients, 28 men and 13 women (ratio 2.1:1) aged between 62 and 91 years (mean 74.2 years, median 71 years), were admitted to 13 of the 17 centres with maxillary fractures. The main cause of injury was falls, accounting for 80% of cases (33 patients), followed by RTA (7 patients), assaults (6 patients), work-related injuries (4 patients), sport-related injuries (1 patient), and other causes (1 patient). As shown in Table 2, the most frequently affected site was the ZMC, followed by Le Fort I fractures, with a prevalence of displaced or comminuted fractures (81%) and a mean FISS score of 1.78 (median 1). Maxillary atrophy at the fracture site, graded from 2 to 6 (mean 4, median 4), was observed in 23 of 41 edentulous patients (56%). Six orbital floor fractures associated with ZMC fractures and four nasal bone fractures were also recorded. Associated injuries were present in 25 out of 41 patients (61%), primarily orthopedic injuries (14 patients), followed by neurological (7 patients), thoracic (3 patients), and ocular injuries (1 patient).

**Table 2:** Edentulous jaws fracture sites and types of management

		Operated			Non operated			
		ND	D	C	ND	D	C	Total
Maxillary	MZC	0	19*	0	3	6	0	28
	Le Fort I-II	2	1	5	3	0	1	12
	Palate	0	1	0	0	0	1	2
Mandible	Body	4	18	0	1	1	0	24
	Symphysis/parasymphysis	3	11	0	1	0	0	15
	Angle	0	6	1	1	0	0	8
	Total	9	56	6	9	7	2	89

* 13 treated with ORIF and 6 treated with a closed approach

*C* comminuted, *D* displaced, *MZC* maxillozygomatic complex, *ND* non-displaced, *ORIF* open reduction and internal fixation

Regarding trauma management, 13 patients (31.7%) with a mean age of 79 years (median 81 years) did not undergo surgery. These patients, who primarily presented with ZMC fractures (Table 2) and had a mean FISS score of 1.86 (median 1), were admitted for observation and maintained on a soft diet for an average duration of 5 days (median: 4 days).

Six patients (14.6%) with a mean age of 71 years (median 69.5 years) underwent closed reduction via a percutaneous hook. All patients presenting with displaced ZMC fractures were observed to have a mean FISS score of 1 (median: 1), and an average hospital stay of 4.5 days (median: 2 days).

The remaining 22 patients (53.7%) with a mean age of 71.6 years (median: 71 years) underwent ORIF with miniplates. More than half of these patients presented with ZMC fractures, followed by Le Fort and palate fractures; 82% of the fractures were displaced. The mean FISS score was 1.9 (median 1) and the average hospital stay was 6.6 days (median: 5.5 days). Fixation of ZMC fractures was performed with single-point fixation in five (38%) patients, double-point fixation in seven (54%) patients, and triple-point fixation in one (8%) patient only. Bone grafting was not performed in any patient with edentulous maxillary fractures. At the 3-month follow-up, no complications or sequelae were reported among the 28 operated patients.

A total of 36 patients, 22 men and 14 women (ratio 1.6:1) aged between 64 and 98 years (mean age 76 years, median: 72.5 years), were admitted to 12 of the 17 centres with mandibular fractures. The main cause of injury was falls, accounting for 67% of patients (24 patients), followed by RTA (5 patients), assaults (3 patients), work-related injuries (3 patients), and other causes (1 patient). The most frequently affected site was the mandibular body, followed by the symphysis/parasymphysis region, as summarised in Table 2. Of the 58 recorded fractures, 44 (76%) were displaced or comminuted. There were 19 single fractures, 13 double fractures, and 4 triple fractures, with a mean FISS score of 2.8 (median: 2). A total of 10 associated condylar fractures in eight patients were also reported. Considering all mandibular fracture sites excluding the condyle, 6 (14%) fracture sites presented no atrophy, 12 (28%) Luhr grade I atrophy, 13 (30%) grade II, and 12 (28%) grade III.

Seven patients (19%) had associated injuries, all of which were orthopaedic.

Regarding treatment, only four (11%) patients with a mean age of 73.2 years (median: 69 years) were admitted for observation and a soft diet. Half of their fractures were non-displaced, the mean FISS score was 1.8 (median 2.5), and the average hospital stay was 4.4 days (median: 4 years).

Overall, 32 patients (89%) with a mean age of 76.2 years (median: 73.5 years) underwent ORIF for their fractures, primarily for fractures of the mandibular body (22 patients) and the symphysis/parasymphysis region (14 patients), as shown in Table 2. Most of these fractures were displaced (41 out of 51, 80%), with a mean FISS score of 2.9 (median 2). There were eight associated condylar fractures, of which four were displaced and four were non-displaced. Fixation was rigid for all fractures (94%) except for two (6%) patients with single fractures: one angle fracture with grade I atrophy and one non-atrophic body fracture, in which a single 1.0 mm plate was used (Table 3). Locking plates were used in only four (13%) patients with double or triple fractures; in three of these patients, plates of thickness between 1.5 mm and 2.0 mm were used, while in one patient a plate of thickness > 2.0 mm was used (Table 4). The prevalent surgical approach was intraoral (26 patients, 81%), including two transmucosal approaches, followed by extraoral approaches (9 for the body, 4 for the symphysis, and 3 for the angle) and one transbuccal approach for an angle fracture. In all six cases where plates of thickness > 2.0 mm were used, the approach was exclusively extraoral (Table 4). None of the 10 condylar fractures were treated with plate osteosynthesis, and no patient underwent bone grafting.

**Table 3:** Overview of edentulous patients with single site mandibular fractures

*N*	Sex	Age (years)	Atrophy*	Site	Approach	Plates 1–1.4 mm (*n*)	Plates 1.5–2 mm (*n*)	Plates > 2 mm (*n*)
1	F	89	No	Body	Intraoral	1	0	0
2	F	76	No	Angle	Transbuccal	0	1	0
3	M	76	No	Symphysis/parasymphysis	Intraoral	2	0	0
4	M	72	No	Symphysis/parasymphysis	Intraoral	2	0	0
5	M	66	I	Symphysis/parasymphysis	Intraoral	1	1	0
6	F	89	I	Body	Intraoral	0	1	0
7	M	72	I	Body	Intraoral	0	1	0
8	M	72	I	Angle	Intraoral	2	0	0
9	F	69	I	Body	Extraoral	0	0	1
10	M	65	I	Angle	Intraoral	1	0	0
11	M	64	I	Symphysis/parasymphysis	Intraoral	2	0	0
1	M	70	II	Angle	Extraoral	0	2	0
13	M	74	II	Symphysis/parasymphysis	Extraoral	0	1	0
14	M	72	II	Body	Intraoral	2	0	0
15	M	73	II	Body	Intraoral	1	1	0
16	M	79	III	Angle	Extraoral	0	0	1
17	F	88	III	Body	Extraoral	0	1	0

* Luhr classification

**Table 4:** Overview of edentulous patients with double and triple mandibular fractures

*N*	Sex	Age (years)	Site(s)	Atrophy*	Approach	Plates 1–1.4 mm (*n*)	Plates 1.5–2 mm (*n*)	Plates > 2 mm (*n*)	Site(s)	Atrophy	Approach	Plates 1–1.4 mm (*n*)	Plates 1.5–2 mm (*n*)	Plates > 2 mm (*n*)
18	M	68	Symphysis/parasymphysis	No	Intraoral	0	2	0	C					
19	F+	71	Body	I	Intraoral	2	0	0	C **+** C					
20	F	89	Body	I	Extraoral	2	0	0	Condyle (head)					
21	M	74	Body	I	Intraoral	0	2	0	Body	I	Intraoral	0	1	0
22	M	71	Symphysis/parasymphysis	I	Intraoral	1	1	0	Angle	None	Intraoral	0	1	0
23	F	88	Body	II	Intraoral	1	1	0	Symphysis/parasymphysis	II	Intraoral	1	1	0
**24**	M	78	Body	II	Intraoral	0	1	0	Symphysis/parasymphysis + C	II	Intraoral	2	0	0
25	F	64	Body	II	Extraoral	0	2	0	Body	II	Extraoral	0	2	0
26	F	83	Body	II	Extraoral	0	1°	0	Body	II	Extraoral	0	1°	0
27	M	69	Symphysis/parasymphysis	II	Intraoral	0	2	0	Condyle (neck)					
28	F	91	Body	III	Intraoral transmucosal	0	1 L°	0	Body +C	III	Intraoral transmucosal	0	1 L°	0
29	M	98	Body	III	Extraoral	0	0	1°	Body	III	Extraoral	0	0	1°
30	F	84	Body	III	Intraoral transmucosal	0	1 L°	0	Symphysis/parasymphysis	III	Intraoral transmucosal	0	1 L°	0
31	F	64	Symphysis/parasymphysis	III	Extraoral	0	0	1 L°	Angle	III	Extraoral	0	0	1 L°
32	F	80	Symphysis/parasymphysis	III	Extraoral	0	1 L°	0	Symphysis/parasymphysis	III	Extraoral	0	1 L°	0

* Luhr classification

° A single plate spanning both mandibular fracture sites was placed for each patient

+ C: associated condyle fracture, not surgically treated; L: locking plate

Statistical analysis showed that displaced or comminuted fracture sites underwent ORIF significantly more frequently than non-displaced fracture sites, both for maxillary (76% vs. 25%, *p* = 0.010, Fisher’s exact test) and mandibular fractures (97% vs. 10%, *p* = 0.026, Fisher’s exact test).

With regard to mandibular atrophy, statistical analysis demonstrated that the distribution of osteosynthesis plate selection was not uniform across atrophy grades. A statistically significant association was observed only between the use of 1.0–1.4 mm thick plates and grade 0–1 mandibular atrophy (adjusted *p* = 0.008, chi-square test with Bonferroni correction).

At the 3-month follow-up among the 32 patients treated with ORIF, intraoral plate exposure was observed in three (9%) patients (involving an angle fracture, a body fracture, and a symphysis/parasymphysis fracture). One (3%) patient experienced inferior alveolar paraesthesia following a displaced parasymphysis fracture. One (3%) patient with a comminuted angle fracture, fixed with a plate of thickness > 2 mm via an external approach, required reoperation due to plate displacement; a longer plate of the same thickness was repositioned, also via an external approach.

## Discussion

A recent meta-analysis reported that the incidence of edentulism in the global elderly population is approximately 35%, with higher percentages of edentulism observed in developing countries [26]. This prospective multi-centre study shows a lower incidence, which is likely attributable to the fact that two-thirds of the centres participating in the WORMAT project are located in developed countries.

While the male predominance and mean age are similar to the findings reported in various articles on maxillofacial trauma in the elderly [1–3, 6–8], edentulous patients with jaw fractures in our study demonstrated a significantly higher incidence of falls than that typically cited in the literature, where the percentages range from 50% [27] to 68% [1]. This discrepancy may be due to the fact that edentulism, as observed in more than two-thirds of the patients in this study, is often associated with bone resorption [28, 29], which weakens specific areas of the jaws such as the mandibular body. Consequently, in patients with severe bone atrophy, even medium- to low-energy trauma can cause a fracture. Furthermore, edentulism may predispose the maxilla to such fractures because, as noted by Crawley et al., “dentition may also provide some protection from fracture by absorbing traumatic forces, which are dissipated by the breaking of the dentition” [17].

As described in several articles [2, 5, 16, 17, 28, 30], surgical treatment in the elderly population is often reserved for displaced fractures, a finding consistent with our observations. However, in line with the report by Gerbino et al. [31], only half of the patients with maxillary fractures in our series underwent ORIF, compared to more than 80% of elderly patients with mandibular fractures. Indeed, in one-third of the patients with maxillary fractures, often presenting with non-displaced ZMC or Le Fort fractures, the decision was made for observation and a soft diet because, especially for maxillary fractures, “there was no functional deficit, and the operation would have been essentially one of aesthetic or cosmetic reconstruction” [31].

Although the numbers in this study are limited due to the rarity of fractures in edentulous patients and a propensity for conservative management in the elderly, a tendency was observed to treat almost all ZMC fractures with open reduction and single- or double-point internal fixation. This is despite recent systematic reviews concluding that three-point fixation provides greater stability and reduces malar asymmetry [32, 33]. Furthermore, in seven out of thirteen patients with ZMC fractures, fixation involved the frontozygomatic buttress and/or the infraorbital rim. As reported by numerous authors [3, 17, 30], maxillary edentulism with consequent progressive atrophy of the alveolar process may result in insufficient bone quantity and quality to achieve stable fixation with plates and screws on the zygomaticomaxillary buttress. Moreover, as noted by Crawley et al. [17], plates placed via an intraoral approach may interfere with the placement of dental prostheses and therefore may require subsequent removal.

Among the centres participating in this project, greater uniformity was observed in the decision to treat all edentulous patients with non-condylar mandibular fractures using ORIF, despite their higher mean age compared to patients with maxillary fractures. The same uniformity was also evident in the choice of fixation type, which was consistently rigid except for two patients, both with single fractures: a non-atrophic body fracture and an angle fracture with grade I atrophy, which were therefore deemed to have sufficient bone volume for non-rigid fixation with plates of thickness < 1.5 mm. In contrast, single plates of thickness ≥ 1.5 mm were used in four out of seven patients where the locking technique was employed, almost exclusively in mandibular fractures with grade III atrophy, as shown in Table 4, thereby utilising a load-bearing principle. The bone of an atrophic mandible, particularly with a vertical height below 10 mm at the fracture site, must not share any occlusal load; instead, the load is borne by the locking plate [9]. Therefore, in line with the literature [9–15] and confirmed by a recent systematic review [34], the greater the degree of mandibular atrophy, the greater the required rigidity of the osteosynthesis and, consequently, the plate thickness. This aligns with the principle summarised as: the smaller the mandible, the larger the plate to be used [9]. Indeed, as reported by Ellis and Price [9], miniplates do not offer adequate resistance against the tensile forces that are generated, particularly at the level of the edentulous mandibular body, despite the lower masticatory forces in these patients compared to dentate individuals. The external approach (or transmucosal in two patients) was always used for the placement of plates > 2.0 mm in fractures with grade III atrophy and in half of those with grade II atrophy. This approach, in agreement with the literature [9, 13–15, 35], allows for adequate exposure of the fracture site, especially in cases of double fractures, while preserving the marginal mandibular branch of the facial nerve, the inferior alveolar nerve (which frequently lies on top of the alveolar crest in atrophic mandibles), and the superior and lingual attachments of the periosteum.

Finally, in contrast to the findings of Shi et al., who recommend ORIF for condylar fractures in edentulous patients with loss of ramus vertical height, none of these fractures were surgically treated in the participating centers. This approach may reflect a risk–benefit consideration in a patient population in which surgical risks may outweigh the benefits of anatomical reduction, particularly given that prostheses can be adjusted in cases of mild changes in the intermaxillary relationship.

The limitations of this study include a relatively small sample size, which underscores the rarity of this type of fracture—a fact also confirmed by the scarcity of publications in the literature, especially concerning edentulous maxillary fractures. Furthermore, the multicentre design implies that the surgical procedures were performed by different maxillofacial surgeons across the various centres participating in the WORMAT project, and the individual surgeon’s experience may have influenced the outcomes. Finally, the relatively brief follow-up period of 3 months may limit the detection of late-onset complications.

The authors hope for future prospective multicentre studies to collect a larger sample of patients and obtain more comprehensive information on the fixation strategies for edentulous jaw fractures, with the aim of establishing shared surgical protocols. Such studies should also ideally include the evaluation of patient-centered outcomes, such as time to return to prosthetic function, quality of life, and pain scores.

## Conclusions

This first prospective multicentre study analyses a population of edentulous elderly patients with jaw fractures, who often present with bone atrophy and whose injuries are primarily caused by falls, with a male predominance. Among the 17 centres participating in the WORMAT project, uniformity was observed in the choice to treat mainly displaced fractures with ORIF, particularly fractures of the mandible, with a clear tendency to employ rigid fixation.

Despite the limitations related to the size of the analysed sample, these data contribute to clarifying therapeutic strategies for this patient population and underline the importance of a treatment approach tailored to the degree of bone atrophy.

## Data Availability

The datasets generated during this study are not publicly available due to privacy requirements, but are available from the corresponding author upon reasonable request.

## References

[1] Possebon APDR, Granke G, Faot F et al (2017) Etiology, diagnosis, and demographic analysis of maxillofacial trauma in elderly persons: A 10-year investigation. J Craniomaxillofac Surg 45:1921–1926. 10.1016/j.jcms.2017.09.002 29054310 10.1016/j.jcms.2017.09.002

[2] Bojino A, Roccia F, Carlaw K et al (2022) A multicentric prospective analysis of maxillofacial trauma in the elderly population. Dent Traumatol 38:185–195. 10.1111/edt.12736 35150461 10.1111/edt.12736

[3] Shi LL, Pudney J, Brangman S, Parham K, Nuara (2023) Head and neck trauma in the geriatric population. Otolaryngol Clin N Am 56:1183–1201. 10.1016/j.otc.2023.05.005 10.1016/j.otc.2023.05.005 37385861

[4] Gugliotta Y, Roccia F, Sobrero F, Ramieri G, Volpe F (2024) Changing trends in maxillofacial injuries among paediatric, adult and elderly populations: a 22-year statistical analysis of 3424patients in a tertiary care centre in Northwest Italy. Dent Traumatol 40:187–194. 10.1111/edt.12904 37915278 10.1111/edt.12904

[5] Kultanen E, Haapanen A, Mustasilta R, Puolakkainen T, Abio A, Thorén H, Snäll J (2025) Aging has occurred rapidly in the facial fracture population – are we ready? Clin Oral Investigations 29:564. 10.1007/s00784-025-06640-7 10.1007/s00784-025-06640-7 PMC12612020 41222599

[6] Zelken JA, Khalifian S, Mundinger GS et al (2014) Defining predictable patterns of craniomaxillofacial injury in the elderly: Analysis of 1,047 patients. J Oral Maxillofac Surg 72:352–361. 10.1016/j.joms.2013.08.015 24139294 10.1016/j.joms.2013.08.015

[7] Cecen S, Ozgenel GY, Ozbek S, Akin S et al (2024) Maxillofacial trauma in geriatric patients in an aging country. J Plast Reconstr Aesth Surg 161–169. 10.1016/j.bjps.2024.05.040 10.1016/j.bjps.2024.05.040 38924894

[8] Brucoli M, Boffano P, Romeo I, Corio C et al (2020) Management of maxillofacial trauma in the elderly: A multicenter study. Dent Traumatol 36:241–246. 10.1111/edt.12536 31863620 10.1111/edt.12536

[9] Ellis E, Price C (2008) Treatment protocol for fractures of the atrophic mandible. J Oral Maxillofac Surg 66:421–435. 10.1016/j.joms.2007.08.042 18280373 10.1016/j.joms.2007.08.042

[10] Melo AR, de Aguiar Soares Carneiro SC, Leal JLF, Vasconcelos BCE (2011) Fractures of the atrophic mandible: case series and critical review. J Oral Maxillofac Surg 69:1430–1435. 10.1016/j.joms.2010.05.078 21216069 10.1016/j.joms.2010.05.078

[11] Novelli G, Sconza C, Ardito E, Bozzetti A (2012) Surgical treatment of the atrophic mandibular fractures by locked plates systems: our experience and a literature review. Craniomaxillofac Trauma Reconstruction 5:65–74. 10.1055/s-0031-1300961 10.1055/s-0031-1300961 PMC3444021 23730420

[12] Flores-Hidalgo A, Altay MA, Atencio IC, Manlove AE, Schneider KM, Baur DA, Quereshy FA (2015) Management of the atrophic mandible: a case series. Oral Surg Oral Med Oral Pathol Oral Radiol 119:619–627. 10.1016/j.oooo.2015.01.016 25782725 10.1016/j.oooo.2015.01.016

[13] Batbayar EO, Bos RRM, van Minnen B (2018) A treatment protocol for fractures of edentulous mandible. J Oral Maxillofac Surg 76:2151–2160. 10.1016/j.joms.2018.04.010 29746839 10.1016/j.joms.2018.04.010

[14] Gerbino G, Cocis S, Roccia F et al (2018) Management of atrophic mandibular fractures: An Italian multicentric retrospective study. J Craniomaxillofac Surg 46:2176–2181. 10.1016/j.jcms.2018.09.020 30333079 10.1016/j.jcms.2018.09.020

[15] Stathopoulos P, Parara E, Krasadakis C et al (2024) Management of the atrophic mandibular fractures: a ten-year prospective study of 48 injuries. Oral Maxillofac Surg 28:1321–1325. 10.1007/s10006-024-01260-z 38778002 10.1007/s10006-024-01260-z

[16] Cooter RD, Dunaway DJ, David DJ (1996) The influence of maxillary dentatures on mid-facial fractures patterns. Br J Plast Surg 49:379–382. 10.1016/s0007-1226(96)90006-5 8881784 10.1016/s0007-1226(96)90006-5

[17] Crawley WA, Azman P, Clark N, Robertson B et al (1997) The edentulous Le Fort fracture. J Craniofac Surg 8:298–307. 10.1097/00001665-199707000-00013 9482055 10.1097/00001665-199707000-00013

[18] Roccia F, Iocca O, Sobrero F et al (2022) World Oral and Maxillofacial Trauma (WORMAT) project: A multicenter prospective analysis of epidemiology and patterns of maxillofacial trauma around the world. J Stomatol Oral Maxillofac Surg 123:e849–e857. 10.1016/j.jormas.2022.05.004 35545192 10.1016/j.jormas.2022.05.004

[19] Luhr HG, Reidick T, Merten HA (1996) Results of treatment of fractures of the atrophic edentulous mandible by compression plating: A retrospective evaluation of 84 consecutive cases. J Oral Maxillofac Surg 54:250–254. 10.1016/s0278-2391(96)90733-8 8600229 10.1016/s0278-2391(96)90733-8

[20] Cawood JI, Howell RA (1988) A classification of the edentulous jaws. Int J Oral Maxillofac Surg 17:232–236. 10.1016/s0901-5027(88)80047-x 3139793 10.1016/s0901-5027(88)80047-x

[21] Bagheri SC, Dierks EJ, Kademani D, Holmgren E, Bell RB, Hommer L, Potter BE (2006) Application of a facial injury severity scale in craniomaxillofacial trauma. J Oral Maxillofac Surg 64:408–414. 10.1016/j.joms.2005.11.013 16487802 10.1016/j.joms.2005.11.013

[22] Ehrenfeld M, Manson PN, Prein J (2012) Principles of internal fixation of the craniomaxillofacial skeleton. Trauma Orthognathic Surg. 10.1097/prs.0000000000000620

[23] Ellis E (2013) Open reduction and internal fixation of combined angle and body/symphysis fractures of the mandible: How much fixation is enough? J Oral Maxillofac Surg 71:726–733. 10.1016/j.joms.2012.09.017 23245520 10.1016/j.joms.2012.09.017

[24] Ellis E (2014) An algorithm for the treatment of noncondylar mandibular fractures. J Oral Maxillofac Surg 72:939–949. 10.1016/j.joms.2013.11.026 24480758 10.1016/j.joms.2013.11.026

[25] Rughubar V, Vares Y, Singh P et al (2020) Combination of rigid and nonrigid fixation versus nonrigid fixation for bilateral mandibular fractures: a multicenter randomized controlled trial. J Oral Maxillofac Surg 78:1781–1794. 10.1016/j.joms.2020.05.012 32589939 10.1016/j.joms.2020.05.012

[26] Roberto LL, Crespo TS, Monteiro-Junior RS, Martins AMEBL et al (2019) Sociodemographic determinants of edentulism in the elderly population: a systematic review and meta-analysis. Gerodontology 36:325–337. 10.1111/ger.12430 31274222 10.1111/ger.12430

[27] Mundinger GS, Bellamy JL, Miller DT, Christy MR et al (2016) Defining population-specific craniofacial fracture patterns and resource use in geriatric patients: a comparative study of blunt craniofacial fractures in geriatric versus nongeriatric adult patients. Plast Reconstr Surg 137:e386–e393. 10.1097/01.prs.0000475800.15221.cd 10.1097/01.prs.0000475800.15221.cd 26818329

[28] Jacobs R (2003) Preoperative radiologic planning of implant surgery in compromised patients. Periodontol 2000 33:12–25. 10.1046/j.0906-6713.2002.03302.x 12950838 10.1046/j.0906-6713.2002.03302.x

[29] Tallgren A (2003) The continuing reduction of the residual alveolar ridges in complete denture wearers: a mixed-longitudinal study covering 25 years. J Prosthet Dent 89:427–435. 10.1016/s0022-3913(03)00158-6 12806317 10.1016/s0022-3913(03)00158-6

[30] Kushner GM, Flint RL (2020) Geriatric and edentulous maxillary and mandibular fractures. From facial trauma surgery: from primary repair to reconstruction. ed. Elsevier, pp 280–281. Chap. 1.21

[31] Gerbino G, Roccia F, De Gioanni PP, Berrone S (1999) Maxillofacial trauma in the elderly. J Oral Maxillofac Surg 57:777–782. 10.1016/s0278-2391(99)90812-1 10416623 10.1016/s0278-2391(99)90812-1

[32] Gadkari N, Bawane S, Chopra R, Bhate K, Kulkarni D (2019) Comparative evaluation of 2-point vs 3-point fixation in the treatment of zygomaticomaxillary complex fractures – A systematic review. J Craniomaxillofac Surg 47:1542–1550. 10.1016/j.jcms.2019.07.009 31395419 10.1016/j.jcms.2019.07.009

[33] Jazayeri HE, Khavanin N, Yu JW, Lopez J, Shamliyan T, Peacock ZS, Dorafshar AH (2019) Fixation points in the treatment of traumatic zygomaticomaxillary complex fractures: A systematic review and meta-analysis. J Oral Maxillofac Surg 77:2064–2073. 10.1016/j.joms.2019.04.025 31132344 10.1016/j.joms.2019.04.025

[34] Bera RN, Tiwari P (2023) Current evidence for the management of edentulous atrophic mandible fractures: A PRISMA-SwiM guided review. Craniomaxillofac Trauma Reconstr 16:317–322. 10.1177/19433875221115585 38047145 10.1177/19433875221115585 PMC10693259

[35] Roccia F, Cena P, Cremona G, Garzino Demo P, Sobrero F (2025) Characteristics and surgical management of bilateral body mandibular fractures: A 23-year experience. J Clin Med 14:160. 10.3390/jcm14010160 10.3390/jcm14010160 PMC11721183 39797243

